# Microscopy Visualization of Carrier Transport in CdSeTe/CdTe Solar Cells

**DOI:** 10.1021/acsami.2c09426

**Published:** 2022-08-24

**Authors:** Chuanxiao Xiao, Chun-Sheng Jiang, Marco Nardone, David Albin, Adam Danielson, Amit H. Munshi, Tushar Shimpi, Walajabad Sampath, Sean Jones, Mowafak M. Al-Jassim, Glenn Teeter, Nancy M. Haegel, Helio R. Moutinho

**Affiliations:** † National Renewable Energy Laboratory, Golden, Colorado 80401, United States; ‡ Department of Physics and Astronomy, Bowling Green State University, Bowling Green, Ohio 43403, United States; § Colorado State University, Fort Collins, Colorado 80523, United States

**Keywords:** carrier transport, CdTe, CdSeTe, solar cell, microscopy, interface, recombination

## Abstract

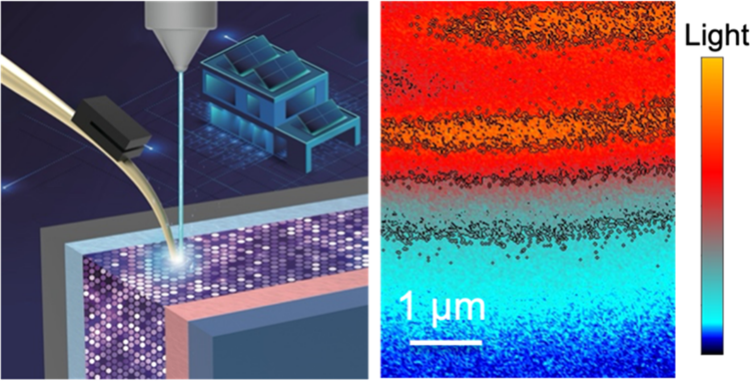

Solar cells are essentially minority carrier devices, and it is therefore of central importance to understand the pertinent carrier transport processes. Here, we advanced a transport imaging technique to directly visualize the charge motion and collection in the direction of relevant carrier transport and to understand the cell operation and degradation in state-of-the-art cadmium telluride solar cells. We revealed complex carrier transport profiles in the inhomogeneous polycrystalline thin-film solar cell, with the influence of electric junction, interface, recombination, and material composition. The pristine cell showed a unique dual peak in the carrier transport light intensity decay profile, and the dual peak feature disappeared on a degraded cell after light and heat stressing in the lab. The experiments, together with device modeling, suggested that selenium diffusion plays an important role in carrier transport. The work opens a new forum by which to understand the carrier transport and bridge the gap between atomic/nanometer-scale chemical/structural and submicrometer optoelectronic knowledge.

## Introduction

In solar cell operation, the light-absorbing material absorbs photons to generate charge carriers, and then carriers are transported to the electron/hole selection parts of the device.^1^ The carrier transport, including both diffusion and drift, at any part of the device is a critical aspect for every solar cell. To better understand device physics and further improve device performance, it is essential to draw a clear picture of the carrier transport process within the device.

Thin-film solar cells are three-dimensionally inhomogeneous in both structure and optoelectronic behaviors; carrier transport investigation remains very challenging. Cadmium telluride (CdTe)-based photovoltaics are the market-leading thin-film solar conversion technology, which can be manufactured quickly and inexpensively. In recent years, the laboratory-based CdTe cell efficiency has improved progressively to 22.1%, and commercial module efficiency has reached 19%.^2,3^ Increasing the device performance without substantially inducing an increase in cost is of great interest. One promising approach is to incorporate selenium (Se) into CdTe absorber materials to make CdSeTe.^4−7^ CdSeTe has a lower band gap, and its incorporation has been shown to increase carrier lifetime and reduce recombination at the front side.^8−12^ On the other hand, CdCl_2_ treatment is ubiquitously used in CdTe-based techniques. The CdCl_2_ treatment applies the CdCl_2_-containing solution or vapor on top of the film and anneals it at a high temperature to allow Cl diffusion, where the CdTe top surface often has higher material quality (larger grains, higher crystallinity, longer lifetime, higher mobility, lower defect density).^13−17^ These factors result in a very complicated device structure with different polycrystalline thin-film layers and interfaces. Further, the stability of the CdTe-based photovoltaic is crucial, especially with large-scale field deployment in recent years. The CdTe device performance degradation could be dominated by fill factor (FF), open-circuit voltage (*V*_oc_), by both *V*_oc_ and FF, or short-circuit current (*J*_sc_), depending on the stress conditions under voltage and/or light biases.^18−25^ The various performance-loss phenomena suggest that excitation associated with charge carrier transport may be different after degradation.

A better understanding of the carrier transport phenomena within real devices will generate knowledge that could possibly result in solar cells with higher efficiencies and greater stability. We have developed a near-field transport imaging (TI) technique to locally characterize charge carrier recombination in thin-film solar cells.^26−28^ Here, we applied the TI technique on the cross section of CdTe-based thin-film solar cells, allowing the direct visualization of carrier transport within the device both before and after degradation. We found that the carrier transport properties were significantly different between pristine and degraded cells, which could possibly be explained by Se interdiffusion toward the back-contact. However, it should be noted that the change of Se gradient is a result of degradation. The detailed degradation mechanism was discussed in our previous publication, that is, an increased electric field near the MgZnO (MZO)/CdSeTe interface due to the loss of MZO doping and/or increase of the conduction band offset spike.^29^ The unique TI technique provided a possibility to image carrier transport inside a device affected by multiple physical mechanisms. Device modeling supports the experimental results and deepens the understanding of carrier transport with complex features in thin-film solar cells.

## Results and Discussion

**Figure 1:**
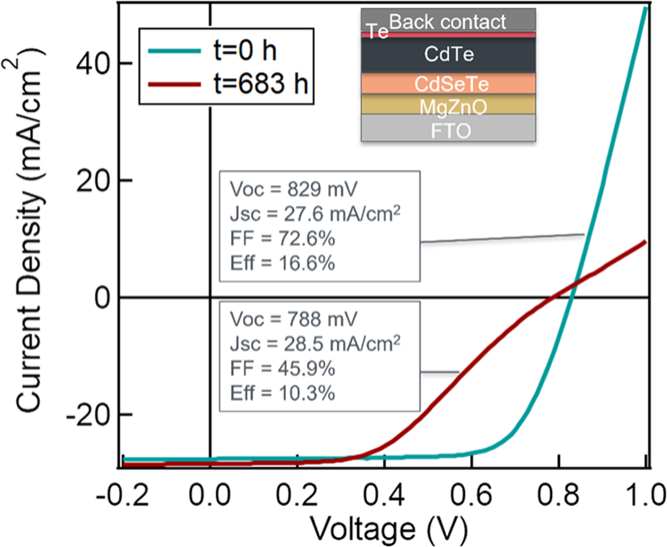
Light *J*–*V* curves of a representative CdSeTe/CdTe device before and after stressing, together with the cell schematic (not to scale).

The cells used in this study had an MZO buffer layer and a graded CdSeTe/CdTe absorber. More details about cell architecture and materials of the devices can be found in ref (6). Mainly, an 800 nm CdSe*_x_*Te_1–*x*_ layer was added to the absorber, and Se diffused into the CdTe layer in the next 1.5–2 μm. Hence, the cell has a graded CdSeTe/CdTe absorber, with a high concentration of Se in the first 800 nm or so. Figure 1 shows the current density–voltage (*J*–*V*) characteristics of a representative cell before and after stressing, together with the cell schematic (more statistical results are found in Tables S1 and S2). The stress condition is 65 °C, 1 sun for about 683 h. Initially, the cell had a *V*_oc_ of 829 mV, a *J*_sc_ of 27.6 mA/cm^2^, an FF of 72.6%, and an efficiency of 16.6%. After stressing, the cell efficiency dropped to 10.3%, with a *V*_oc_ of 788 mV and an FF of 45.9%. The *J*_sc_, however, slightly increased to 28.5 mA/cm^2^. Such degradation here is irreversible. Note that the stress conditions may be extreme for such a device architecture; the results shown here were performed on cells using a porous graphite contact. Cell reliability has subsequently been improved by replacing this with a sputtered metal back-electrode.^30^ Also, after stressing, the *J*–*V* curve showed a “roll-under” characteristic, as well as increased series resistance, which is possibly due to a conduction band offset at the MZO/CdSeTe interface.^7^ The main change in cell performance was FF with a prominent drop. The decrease of FF could point to a degradation at the interface, which should significantly change the carrier recombination process. It is of great interest to map the charge motion and collection in the direction of relevant carrier transport and to understand the cell operation and degradation.

**Figure 2:**
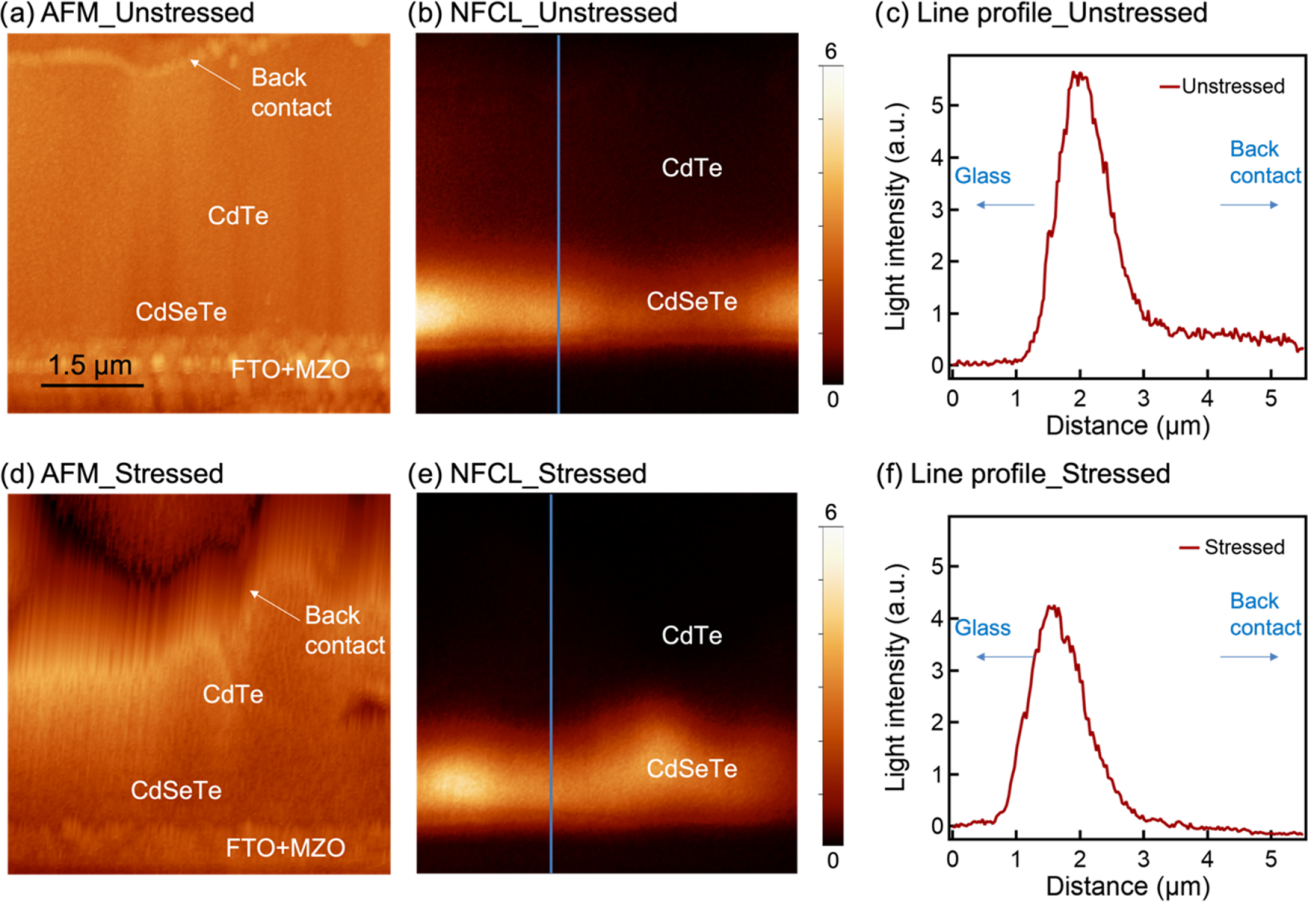
Near-field cathodoluminescence results. (a) atomic force microscopy (AFM) of the unstressed CdSeTe/CdTe device; (b) NF-CL mapping of the unstressed CdSeTe/CdTe device; (c) light intensity profile of NF-CL marked in panel (b); (d) AFM of the stressed CdSeTe/CdTe device; (e) NF-CL mapping of the stressed CdSeTe/CdTe device; and (f) light intensity profile of NF-CL marked in panel (e). The AFM data is shown to help visualize where the NF-CL data is coming from. The luminescence intensity is plotted on a linear scale ranging from 0 to 6. The line profiles start from the glass side toward the back-contact side, as indicated by the arrows.

We first performed near-field cathodoluminescence (NF-CL) measurements on the cross-sectional surface of unstressed and stressed cells, and the results are shown in Figure 2. The NF-CL technique is similar to the conventional CL mapping; see the Methods section for technical details. The front-contact, absorber, and back-contact can be distinguished in the topography images. These NF-CL measurements should be in a high-injection level with an electron beam condition of 20 kV, 1.5 nA. High-injection conditions make the diffusion length of the materials to be position-dependent, and Fermi statistics must be used to accurately evaluate the excess carrier density. In such a circumstance, the diffusivity, lifetime, and surface recombination may vary with injection. It is difficult to unambiguously determine the diffusion length and its correspondence to the electron beam conditions. Also, in high-injection conditions, the built-in electric field is screened due to the large carrier concentration. The measured carrier transport profiles may not mirror the conditions under AM1.5 solar illumination. However, we measured two devices and compared the difference in their carrier transport profiles. In the NF-CL images, the cells both showed a bright Se-rich CdSeTe layer, consistent with the previously reported CdSeTe with a strong CL signal, which could be a result of its high carrier lifetime.^12^ The line profiles showed that unstressed CdSeTe had about 1 order of magnitude brighter CL signal than the CdTe materials. And we observed slightly lower luminescence intensities for both CdSeTe and CdTe in the stressed cell. The qualitative results suggest that new defects formed during stressing and led to higher nonradiative recombination. Note that the NF-CL signal may be affected by surface recombination, but the surface recombination should be similar, as the samples went through the same polishing and annealing procedure (see the Methods section and Figure S1 for another set of samples). The minimal decrease of the NF-CL signal indicates that material degradation is not the major problem in the degradation, which is consistent with the cell performance change observed in Figure 1 that FF is the dominant degradation factor, while *V*_OC_ and *J*_sc_ did not change significantly.

**Figure 3:**
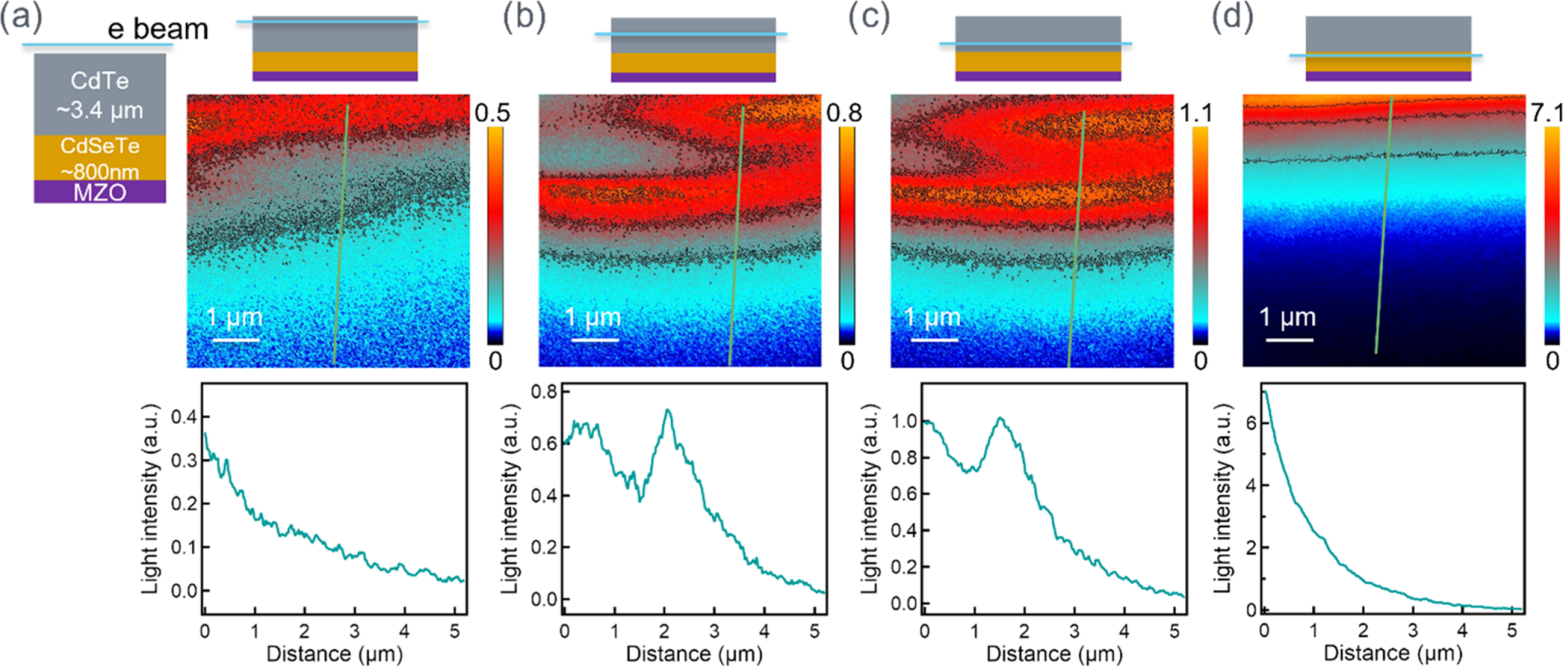
Mapping carrier transport inside the unstressed CdSeTe/CdTe device, with the electron beam fixed in a line scan to excite carriers in different regions, its corresponding transport imaging, and a representative line profile marked in green line (not to scale). (a) Excitation in the CdTe layer; (b) excitation in the CdSeTe intermixture region; (c) excitation in the CdSeTe intermixture region, closer to CdSeTe; and (d) excitation in the CdSeTe layer. The TI color plots extend from the electronbeam line location at the top of the image down toward, and sometimes into, the substrate.

We mapped the carrier transport within the device, with the electron beam generation at different locations on its cross-sectional surface. It should be noted that carrier transport involves both carrier diffusion and drift. Carriers diffuse due to concentration gradient into the bulk and toward carrier “sinks” (defect or surface), which involves both radiative and nonradiative recombination mechanisms. Carriers can drift in built-in electric fields and lead to charge separation. In this paper, the measured “carrier transport” light profile/decay is a detectable portion of radiative carrier recombination. We set the electron beam in a line excitation mode and parallel to the layers; while the carriers can diffuse or drift, the decay will only be seen perpendicular to the carrier generation line. As shown in Figure 3, with carriers generated in different locations, the measured carrier transport light profile/decay can be significantly different. With carrier excitation in CdTe close to the back-contact, we observed a smooth decay: the highest luminescence near the excitation, and as carriers moved away, electrons and holes recombined radiatively and light was collected by the near-field scanning optical microscopy (NSOM) probe (Figure 3a). And in Figure 3d, when the carrier generation is within the CdSeTe region close to the substrate, the carrier transport behavior was similar, only with a much brighter initial luminescence. This is because CdSeTe has a much brighter CL than CdTe, consistent with the results shown in Figure 2. While the carrier excitation was close to the CdTe/CdSeTe intermixture materials, we initially observed the signal decay and then an increase in the light intensity on the CdSeTe layer (Figure 3b,c). These results indicated a complex carrier transport behavior across the device, where the TI technique provided a direct visualization of such a physics picture on a microscopic level.

The interesting carrier transport profiles in Figure 3b,c can be explained by the combined effects of radiative recombination behavior in CdTe and CdSeTe materials, interfacial recombination, and built-in junction. The carrier transport can be separated into three regions: (1) Te-rich region, (2) CdTe/CdSeTe intermixture region, and (3) Se-rich region. In region 1 (mostly CdTe), electron–hole (e-h) pairs were generated when an electron beam interacted with the materials. The start point always has a bright signal proximity to the point of generation and maximum recombination. Then, carriers diffuse away and recombine, both radiatively and nonradiatively. While the detailed injection-level determination requires rigorous modeling, to simplify the discussion, we assumed a high injection in this depletion region and did not consider the electric field effect. In region 2 (intermixture), however, when the carrier transport approached the materials with a higher Se concentration, the narrower band gap and the less nonradiative recombination centers^11,31^ could yield a higher light signal. Hence, a higher luminescence is possible, even with carrier diffusion and the monotonously decreasing carrier concentration. Finally, in region 3 (mostly CdSe_0.16_Te_0.84_), the carriers continued to diffuse and drift inside the CdSe_0.16_Te_0.84_ layer with a higher CL signal. Therefore, we first observed a carrier decay, then a higher TI signal when approaching CdSe_0.16_Te_0.84_, and finally the carrier decay toward the substrate. Note that the second peak may not exactly correlate to the CdSe_0.16_Te_0.84_ layer because the light intensity is convoluted by carrier recombination and material properties. The condition in Figure 3c is similar, only with carrier generation closer to the CdSe_0.16_Te_0.84_ layer, leading to a smaller dip at *x* ∼ 1 μm. Note that the inconsistencies in Figure 3b,c could be due to nonuniform film properties. However, it does not affect the trend that the signal first decays and then increases in the light intensity. In Figure 3b,c, the carrier transport decay profiles are not a single decay, regardless of stronger initial light intensity or a weaker second peak. More decay profiles on different locations and different samples are shown in Figures S2–S5.

**Figure 4:**
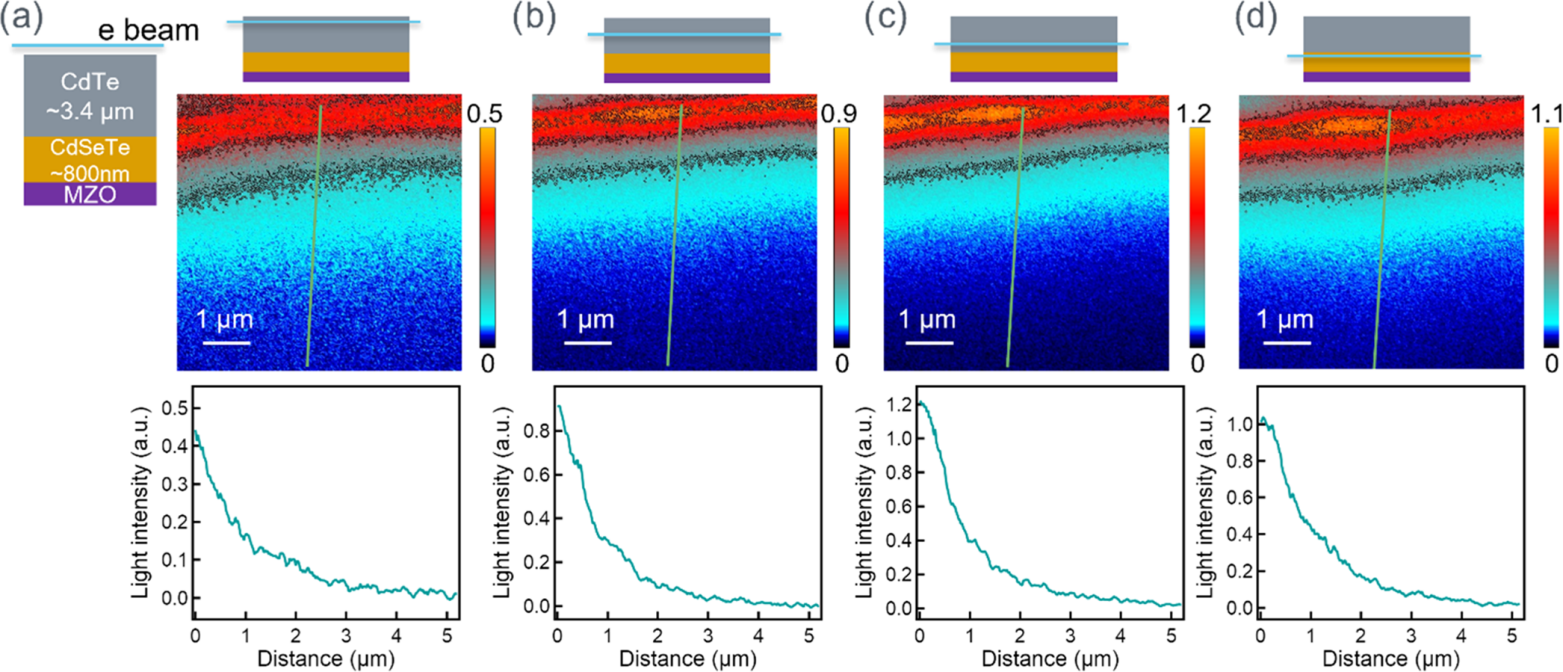
Mapping carrier transport inside the stressed CdSeTe/CdTe device, with an electron beam fixed in a line scan to excite carriers in different regions, its corresponding transport imaging, and a representative line profile marked in green line (not to scale). (a) Excitation in the CdTe layer; (b) excitation in the CdSeTe intermixture region; (c) excitation in the CdSeTe intermixture region, closer to CdSeTe; and (d) excitation in the CdSeTe layer.

**Figure 5:**
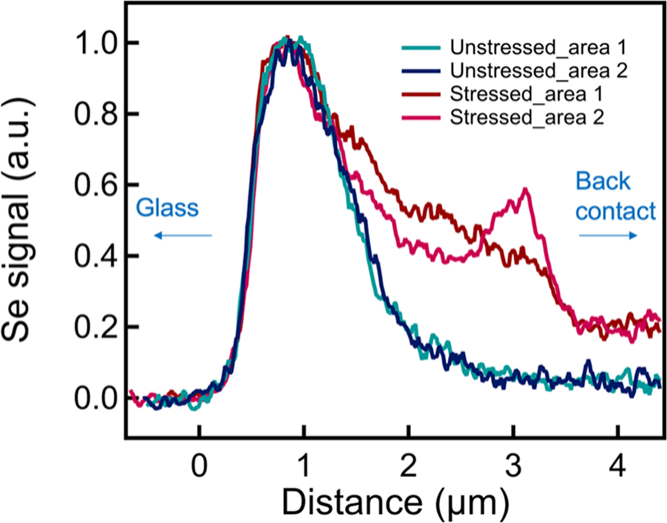
Selenium line profiles on multiple locations of unstressed and stressed cells. We normalized the maximum Se signal to better compare its distribution. The line profiles start from the glass side toward the back-contact side, as indicated by the arrows.

In contrast, from the stressed device, with similar carrier generation locations, we did not observe the dual peak feature in the TI signal. The results from the stressed sample are shown in Figure 4. Similar to the results from the unstressed device, when carrier generation was in the CdTe or CdSeTe layers, the carrier transport profile decayed smoothly, with no effect from the CdTe/CdSeTe intermix. The slight nonuniform signal at the carrier generation line should reflect different material qualities. However, when the carrier generation was near the CdSeTe/CdTe interface, the carrier decays were also smooth, with no significant dip. Also, we found a different Se distribution by scanning electron microscopy (SEM) energy-dispersive X-ray spectroscopy (EDS) on multiple locations of pristine and degraded cells (Figure S6). The Se line profiles, as shown in Figure 5, indicate that the stressed cell has Se diffused further to the back-contact side, which qualitatively agrees with the wider-spreading bright CdSeTe region in Figure 2.

**Figure 6:**
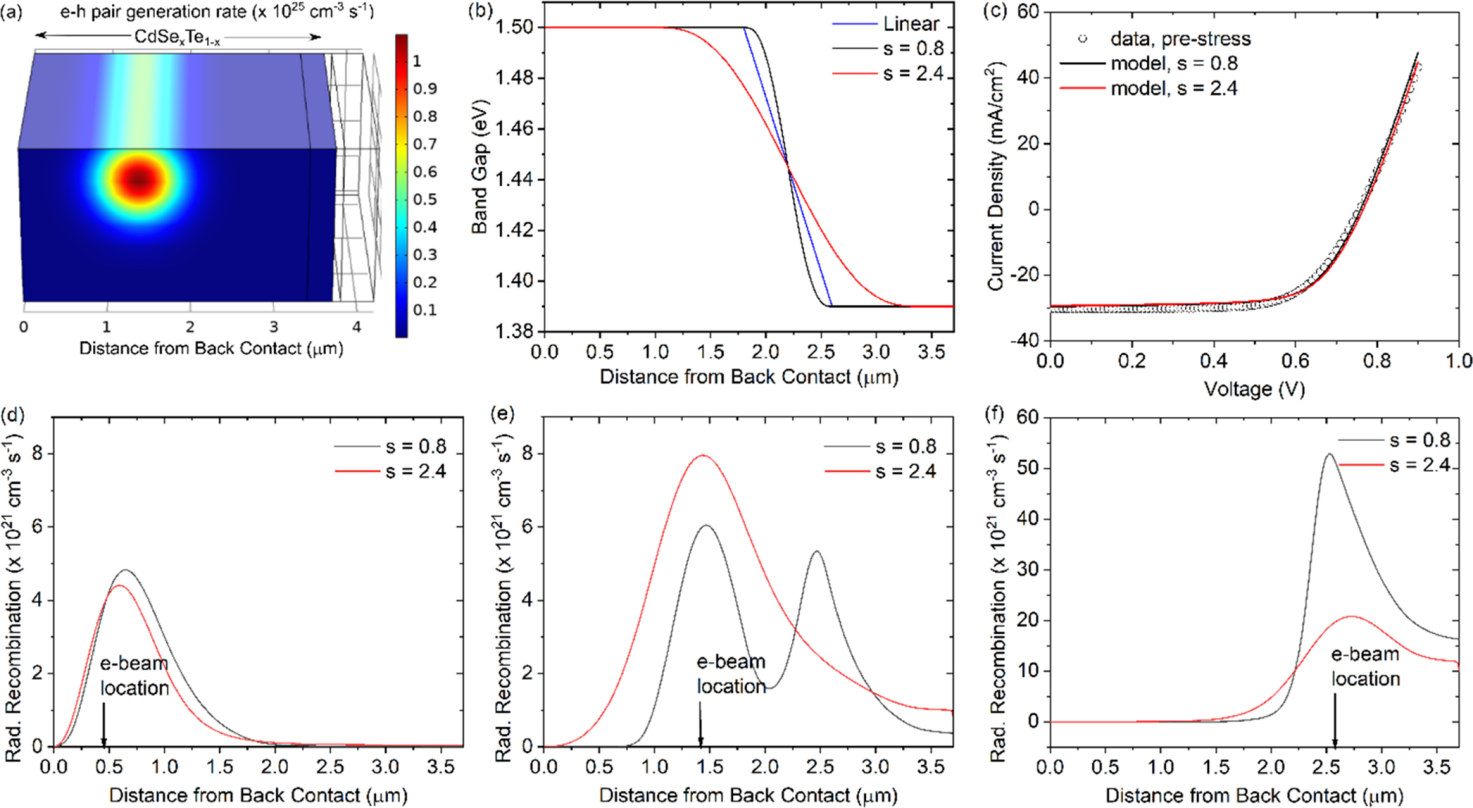
Device modeling results. (a) 3D rendering of 2D TI simulation showing the e–h pair generation rate at the electron beam location *d* = 1.4 μm (excitation in MZO and SnO_2_ not shown); (b) CdSeTe band-gap smoothing variations; (c) simulated *J*–*V* curves and comparison with experimental data for a typical device; and (d–f) radiative recombination rate profiles with electron beam excitation at different locations relative to the distance to back-contact.

To better understand the complex carrier transport, we employed two-dimensional device modeling to simulate the unique carrier transport behavior. The simulations used the COMSOL Multiphysics software to solve the coupled Poisson and electron/hole continuity equations based on the finite element method.^32^Figure 6a illustrates how we modeled transport imaging experiments. The electron beam line scan creates a cylindrical electron–hole pair generation region with a Gaussian cross section of approximately 1 μm in diameter, depending on the beam parameters (see the Methods section). The electron beam location is scanned along the absorber layer from the back to contact to the front, following the TI experimental procedures, while the radiative recombination rate is calculated as a function of the distance from the back-contact for each beam location. The device stack and parameter settings are based on published data;^6,33−35^ details are provided in the Methods section and Table S3. We set the CdSeTe and CdTe to be CdSe*_x_*Te_1–*x*_, where *x* = 0 makes CdTe materials with a band gap of 1.50 eV and *x* = 0.16 makes CdSe_0.16_Te_0.84_ materials with a band gap of 1.39 eV.^36^ For simplicity, the initial model had a linearly graded band gap between the CdTe and CdSe_0.16_Te_0.84_, as shown by the blue line in Figure 6b. Note that the electron affinity was also position-dependent, such that the band-gap grading was entirely accommodated by the variation of the conduction band.^36^

Upon simulating the TI experiments, we noted that the second peak observed in the experiments (Figure 3b,c) was reproduced at the point where the band-gap grading reached a minimum (2.6 μm from the back-contact in Figure 6b). To further test this hypothesis, we varied the band-gap grading using a smoothing parameter (*s*), which determined the length of the transition zone between the high (1.5 eV) and low (1.39 eV) band-gap regions. Note that the modeling aims to capture the most important physics, rather than attempting to model the exact band-gap grading. For the unstressed and stressed devices, band-gap smoothing was set to *s* = 0.8 and 2.4, respectively (Figure 6b). In Figure 6d–f, we plot the radiative recombination profiles along the CdSe*_x_*Te_1–*x*_ layer, with electron beam excitation in three different regions. When the electron beam was located close to the back-contact (*d* = 0.4 μm; Figure 6d), only one peak was observed in the carrier decay. The maximum emitted light intensity was slightly displaced from the excitation point due to interaction of the generation region with the back-contact. When the electron beam was near the CdSe*_x_*Te_1–*x*_ intermix region (*d* = 1.4 μm), the radiative recombination profile of the unstressed sample exhibited two peaks at *d* ≈ 1.4 and 2.6 μm. In contrast, the stressed sample, with band-gap smoothing *s* = 2.4 in the CdSe*_x_*Te_1–*x*_ intermix region, showed only one broader peak at *d* ≈ 1.4 μm. When the electron beam excitation was at the very edge of the CdSe*_x_*Te_1–*x*_ intermix region, the double peak disappeared again on both samples. Note that the magnitude of radiative recombination increases in Figure 6d–6f as the band gap decreases. These profiles are qualitatively consistent with TI and EDS experimental results, indicating that band-gap smoothing due to Se diffusion could be an explanation for the interesting carrier transport and recombination profiles. Investigation of band edge diagrams under electron beam excitation indicates that slight fluctuations in the band edges for *s* = 0.8 result in electron density profiles with the same characteristic double peak as the TI data (see Figure S7). When *s* = 2.4, the band fluctuations are less pronounced and the double peak in carrier density is no longer present.

We also simulated the impact of increasing the band-gap gradient on device performance. Figure 6c shows the calculated *J*–*V* curves for the baseline device with *s* = 0.8 and 2.4. Only a minor reduction in *J*_sc_ and an increase in *V*_oc_ were observed due to the larger band gap near the CdSeTe/MZO interface with *s* = 2.4. Our recent work^29^ reported that the significant FF loss observed after stress was likely due to a decrease in the MZO layer doping or an increase in the conduction band offset. Therefore, the related band-gap smoothing does not appear to be a critical factor in the degradation mechanism. The Se migration by itself does not affect cell performance, but rather, it is a result of light and heat stressing, which alters carrier transport within the device.

Carrier transport is a key aspect of device operation, but the highly inhomogeneous nature of thin-film solar cells makes it difficult to draw a clear physical picture of the complicated carrier transport phenomenon. This work presents measurements on pristine and degraded devices, from macroscale electrical behavior to microscopic chemistry and carrier transport properties, together with numerical simulation. The presented TI technique is a powerful tool for the direct visualization of carrier transport with local variations and the effect of interfacial recombination on a microscopic scale, despite the following limitations: (1) difficult sample preparation and the need for sample encapsulation and fine polishing; (2) charging issue can be severe if there is a lack of sufficient grounding; and (3) other common problems in electron beam-based techniques, such as complicated analysis and long acquisition time. The TI technique, combined with numerical modeling, is expected to open a new forum in which to understand the device physics and bridge the gap between atomic/nanometer-scale chemical/structural and submicrometer optoelectronic knowledge.

## Conclusions

We have applied a near-field transport imaging technique to study localized carrier transport inside CdSeTe/CdTe devices. With the electron beam fixed in a line parallel with the thin-film layers and excitations in different regions, the carrier transport properties show different features inside the device. The carrier transport profiles are a combined effect of material band gaps, bulk radiative and nonradiative recombination, and interfacial recombination. From the unstressed device, the carrier transport exhibits an interesting dual peak when excitation is in the CdSeTe intermixture region. After light and heat stressing in the lab, the dual peak feature disappears, indicating that the carrier transport process is different inside the device. With the aid of device modeling, we incorporated broader band-gap grading in the stressed device, possibly due to Se diffusion (as observed by EDS), to explain the changes in carrier transport decay features. The powerful TI technique, combined with device modeling, shows a unique capability to visualize and quantify carrier transport in highly inhomogeneous polycrystalline thin-film solar cells. The characterization tool can both advance and obtain new fundamental knowledge about the photovoltaic materials and devices, as well as provide a deeper understanding to engineer and improve device performance and stability.

## Methods

### Samples

The detailed cell architecture and materials of the devices can be found in refs (6, 37, 38). The substrate used here is NSG TEC10 soda lime glass coated by fluorine-doped tin oxide (FTO). A 100 nm MgZnO layer was deposited by RF sputtering using a 23 wt % MgO/77 wt % ZnO target. A layer of CdSe*_x_*Te_1–*x*_ was deposited, followed by a CdTe layer by close-spaced sublimation (CSS), forming an 800 nm CdSe*_x_*Te_1–*x*_ layer and a 3.4 μm CdTe layer. Then, an aggressive CdCl_2_ treatment was performed to promote interdiffusion between the CdSe*_x_*Te_1–*x*_ and CdTe layers. Thereafter, a CuCl_2_ treatment was performed to form a Cu-doped back-contact. A 20 nm Te film was evaporated and finally followed by a spray of carbon and nickel paint in a polymer binder to complete the back-electrode.

Cell stressing was conducted at 65 °C at approximately 1 sun irradiance by a xenon lamp. The cells were under an open circuit during stressing. The cell was stressed for 683 h.

### *J*–*V* Characterizations

*J*–*V* curves were measured under 1 sun (AM1.5 G) light intensity from a class AAA Oriel Sol3A solar simulator. The measurements were performed with a four-point probe setup configured for superstrate cells. The *J*–*V* scans were at a speed of ∼3 V/s from −0.25 to 2 V bias with a 6.5 mV step and a dwell time of 2 ms.

### Sample Preparation

We encapsulated the cell with a thin piece of glass with silver epoxy. The cells went through a mechanical polishing with down to 1 μm grit, followed by an ion milling process at room temperature to ensure a flat cross-sectional surface and remove possible mechanical polishing damage.^39^ Finally, the cells were annealed at 250 °C for 5 min to passivate the possible surface damage by the previous polishing steps.^40^

### TI, NF-CL, and EDS Techniques

The TI and NF-CL measurements were performed on the same NSOM system (Nanonics Multiview 2000), with carrier excitation by the electron beam from a field-emission Nova 630 SEM. The measurements were conducted at room temperature. The NSOM probe used in this work has an aperture diameter of 300 nm; the probe collects sample luminescence in the near field but also scans topography. Light emitted from the sample was detected by a silicon detector with 800 nm long-wavelength pass filters (see Figure S8 for an example of the probe landing on the cross-sectional surface of a CdSeTe/CdTe cell). No signal from the SiO_2_ optical fiber was transmitted due to the filters. In TI measurements, the electron beam is scanned in a line parallel to the film surface with a speed of 3 orders of magnitude faster than the NSOM probe scanning; hence, the carrier generation can be considered in a steady state. The NSOM probe collects panchromatic light emitted from the sample. The scan direction starts from the carrier generation region and stops with no luminescence detection. NF-CL is similar to “conventional” CL; both technologies measure the sample cathodoluminescence generated by the electron beam. The difference is the way of signal collection. In “conventional” CL, the electron beam moves from pixel to pixel for carrier generation, and a parabolic mirror collects the emitted sample luminescence in the far field. The CL signal includes light from both the initial carrier generation point and all of the resulting carrier diffusion and recombination. While in NF-CL, the electron beam is fixed and the sample moves instead, the luminescence is collected in a near field by an NSOM probe located near the sample surface. The distance between the electron beam generation point and the NSOM probe is ∼0.8 μm; hence, the collected signal includes both the initial carrier generation and the resulting light after carrier transport to the location of the NSOM probe. NF-CL and CL generate similar luminescence maps that reflect the material properties. We used NF-CL because it can be done directly on our TI setup. The NF-CL capability makes the TI setup more versatile. And it is more convenient to probe the area of interest consecutively with TI scans.

Both TI and NF-CL scans use a 20 kV, 1.5 nA electron beam for carrier generation. One mapping contains 256 × 256 pixels, with a probe dwell time of 8 ms on each point. Note that no carbon deposition contamination affects the NF-CL and further TI analysis. We performed the measurements on two consecutive scans, but the results were very reproducible (Figure S9). The spatial resolution can be affected by a few factors: probe aperture, electron beam (the signal generation volume), and light intensity excited from materials. The key issue is to detect light from materials. If the materials have bright luminescence (high radiative recombination coefficient B), then a smaller probe aperture and lower electron beam voltage and current can be used. For the aperture size, the light signal varies as (*d*/λ)^4^, where d is the diameter of the probe and λ is the wavelength of the emitted radiation. The larger probe aperture collects significantly higher intensity of light signal; on the other hand, it can be challenging to collect light with a very small probe aperture (e.g., 100 nm). And the lower electron beam voltage and current have smaller signal generation volume, allowing for a higher resolution. In this case, the spatial resolution is estimated to be 100 nm, which means the film needs to be thicker than 100 nm to be distinguished. The EDS measurements were also performed on the same SEM.

### Device Modeling

Numerical simulations were performed using COMSOL Multiphysics software v5.6. The coupled semiconductor transport equations were solved using the finite element method. A list of the parameters and their values are provided in Table S3. The input parameters were based on published data and with the fitting of cell *J*–*V* data. Based on our previous results for these devices, a region of slightly n-doped CdSeTe was included within 300 nm of the MZO layer to form a buried homojunction.^29^ Recombination processes, both radiative and nonradiative, were included in the bulk and at the CdSeTe/MZO interface. Band-gap grading was included, as discussed in the Results and Discussion section. Boundary conditions were Schottky contacts specified by surface recombination velocities and barrier heights. The TI simulation emulated the experimental geometry with a 2D model of the device cross section assumed to extend infinitely in the third dimension. The electron beam line formed a cylindrical e–h pair generation region with a Gaussian cross section dependent on the e-beam current, *I*_b_, and energy, *E*_b_.^42,43^ Models used *I*_b_ = 1 nA and *E*_b_ = 20 keV, resulting in a generation region diameter of about 1 and 0.4 μm depths of peak generation below the surface. As shown in Figure 6a, the maximum generation rate is on the order of 10^25^ cm^–3^ s^–1^, which is about 3 orders of magnitude greater than 1 sun light intensity.

## Supporting Information Available

Cell performance and statistical results; more NF-CL results on another set of samples; more decay line profiles showing that the carrier transport of unstressed device is not a single decay; EDS locations; band edges and electron density profiles; SEM image showing how the probe lands on the cross-sectional surface; consecutive NF-CL images showing reliable measurements; and baseline device model parameters (PDF)
