# Supplementary material for: Microscopy Visualization of Carrier Transport in CdSeTe/CdTe Solar Cells
Source: ACS Appl Mater Interfaces. 2022 Aug 24;14(35):39976–84. doi: 10.1021/acsami.2c09426 (PMC9460435; doi:10.1021/acsami.2c09426)
Supplement: Supplementary file 1 — am2c09426_si_001.pdf [file am2c09426_si_001.pdf]

Supporting Materials

Microscopy Visualization of Carrier Transport in CdSeTe/CdTe Solar Cells

Chuanxiao Xiao,[1*] Chun-Sheng Jiang,[1] Marco Nardone,[2] David Albin,[1] Adam Danielson,[3] Amit H. Munshi,[3] Tushar Shimpi,[3] Walajabad Sampath,[3] Sean Jones,[1] Mowafak M. Al-Jassim,[1] Glenn Teeter,[1] Nancy M. Haegel,[1] and Helio R. Moutinho[1]

[1]National Renewable Energy Laboratory, Golden, Colorado 80401, USA

[2]Department of Physics and Astronomy, Bowling Green State University, Bowling Green, Ohio 43403, USA

[3]Colorado State University, Fort Collins, Colorado, 80523, USA

*Present affiliation: 1. Ningbo Institute of Materials Technology and Engineering, Chinese Academy of Sciences, Ningbo City, Zhejiang Province, 315201, China

2. Ningbo New Materials Testing and Evaluation Center CO., Ltd, Ningbo City, Zhejiang Province, 315201, China

Corresponding author: nick.cxiao@gmail.com.

Table S1. Cell performance of unstressed and stressed devices.

Cell	Unstressed				Stressed			
	V_{oc} (mV)	J_{sc} (mA/cm^2)	FF (%)	Eff. (%)	V_{oc} (mV)	J_{sc} (mA/cm^2)	FF (%)	Eff. (%)
1	829.2	27.6	72.6	16.6	787.5	28.5	45.9	10.3
2	828.6	27.2	74.4	16.8	786.6	28.3	47.9	10.7
3	829.2	27.8	75.0	17.3	784.5	27.7	56.8	12.4
4	829.9	27.7	72.4	16.6	789.9	28.8	43.1	9.8
5	829.3	27.4	74.8	17.0	790.3	27.6	46.9	10.2
6	828.4	27.2	75.4	17.0	790.7	26.9	51.8	11.0
7	829.5	28.4	70.3	16.5	792.0	28.6	35.2	8.0
8	829.4	28	73.9	17.1	792.6	29.4	42.4	9.9
9	827.9	27.2	75.2	16.9	792.9	28.5	50.2	11.3
10	829.2	28.4	72.8	17.1	795.9	28.8	45.3	10.4
11	830.4	28.2	74.3	17.4	794.8	29.7	47.4	11.2
12	829.1	27.9	75.7	17.5	792.6	29.1	51.5	11.9
13	750.4	29.1	66.4	14.5	717.9	29.0	51.0	10.6
14	752.1	29.9	64.1	14.4	723.9	29.4	46.3	9.9
15	763.4	32.0	66.9	16.4	728.4	29.8	38.1	8.3
16	756.2	30.3	67.6	15.5	719.0	30.7	41.9	9.2

Table S2. Cell performance change and statistical results of unstressed and stressed devices.

Cell	Absolute change in performance				Change in performance relative to t=0 h			
	ΔV_{oc} (mV)	ΔJ_{sc} (mA/cm^2)	ΔFF (%)	ΔEff. (%)	ΔV_{oc} (%)	ΔJ_{sc} (%)	ΔFF (%)	ΔEff. (%)
1	-41.7	0.9	-26.7	-6.3	-5.0	3.3	-36.8	-38.0
2	-42.0	1.1	-26.5	-6.1	-5.1	4.0	-35.6	-36.3
3	-44.7	-0.1	-18.2	-4.9	-5.4	-0.4	-24.3	-28.3
4	-40.0	1.1	-29.3	-6.8	-4.8	4.0	-40.5	-41.0
5	-39.0	0.2	-27.9	-6.8	-4.7	0.7	-37.3	-40.0
6	-37.7	-0.3	-23.6	-6.0	-4.6	-1.1	-31.3	-35.3
7	-37.5	0.2	-35.1	-8.5	-4.5	0.7	-49.9	-51.5
8	-36.8	1.4	-31.5	-7.2	-4.4	5.0	-42.6	-42.1
9	-35.0	1.3	-25.0	-5.6	-4.2	4.8	-33.2	-33.1
10	-33.3	0.4	-27.5	-6.7	-4.0	1.4	-37.8	-39.2
11	-35.6	1.5	-26.9	-6.2	-4.3	5.3	-36.2	-35.6
12	-36.5	1.2	-24.2	-5.6	-4.4	4.3	-32.0	-32.0
13	-32.5	-0.1	-15.4	-3.9	-4.3	-0.3	-23.2	-26.9
14	-28.2	-0.5	-17.8	-4.5	-3.7	-1.7	-27.8	-31.3
15	-35.0	-2.2	-28.8	-8.1	-4.6	-6.9	-43.0	-49.4
16	-37.2	0.4	-25.7	-6.3	-4.9	1.3	-38.0	-40.6
**Avg.**	**-37.0**	**0.4**	**-25.6**	**-6.2**	**-4.6**	**1.5**	**-35.6**	**-37.5**
**Std.**	**4.0**	**0.9**	**5.1**	**1.2**	**0.4**	**3.2**	**7.0**	**6.8**

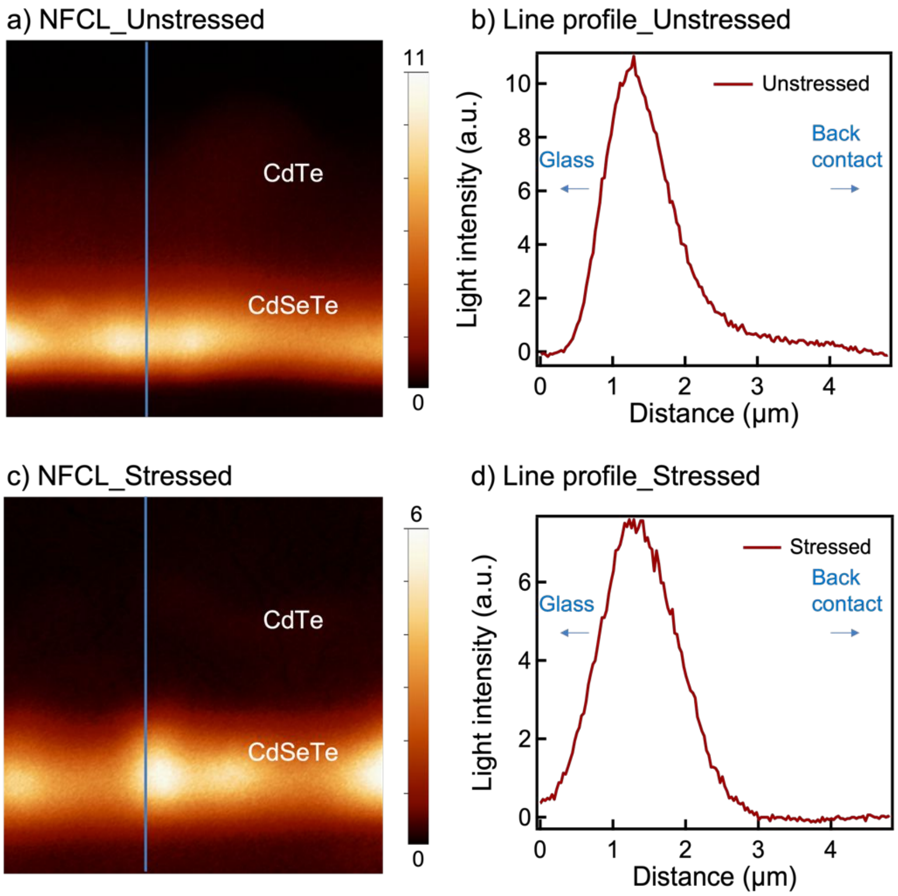

Figure S1. Near-field cathodoluminescence results. a) unstressed CdSeTe/CdTe device; b) light intensity profile of NF-CL marked in a); c) stressed CdSeTe/CdTe device; d) light intensity profile of NF-CL marked in c).

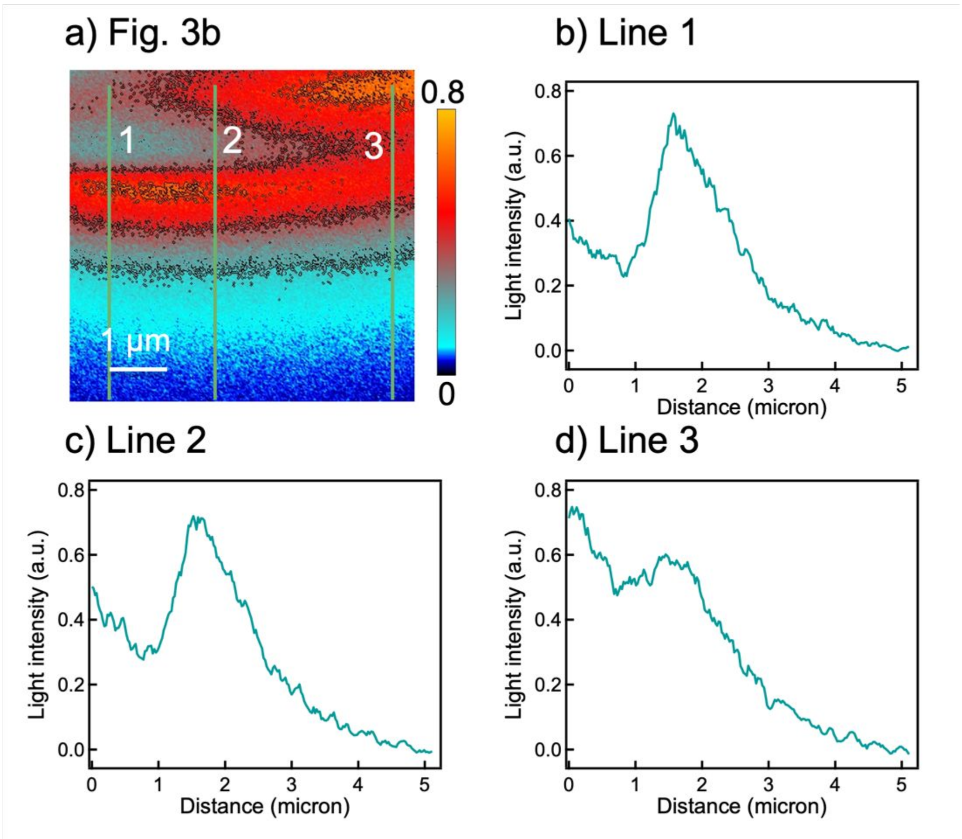

Figure S2. More decay line profiles in Figure 3b, showing the carrier transport decay profiles are not a single decay.

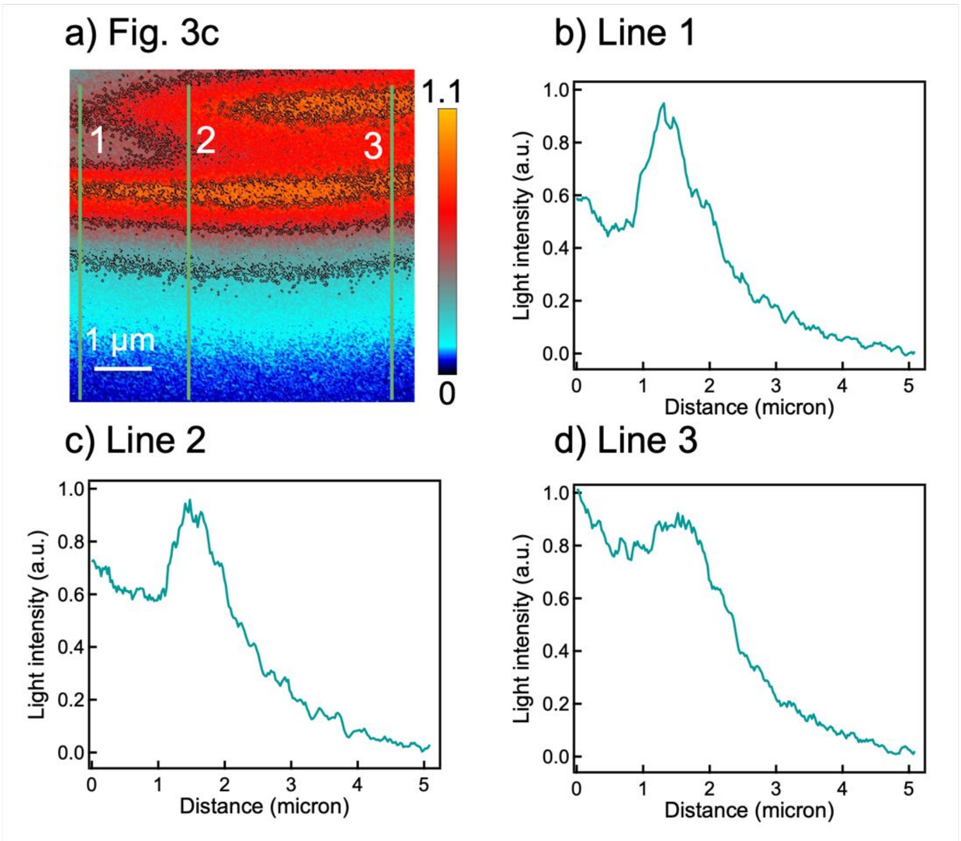

Figure S3. More decay line profiles in Figure 3c, showing the carrier transport decay profiles are not a single decay.

On another unstressed device, we show the dual peak feature is reproducible:

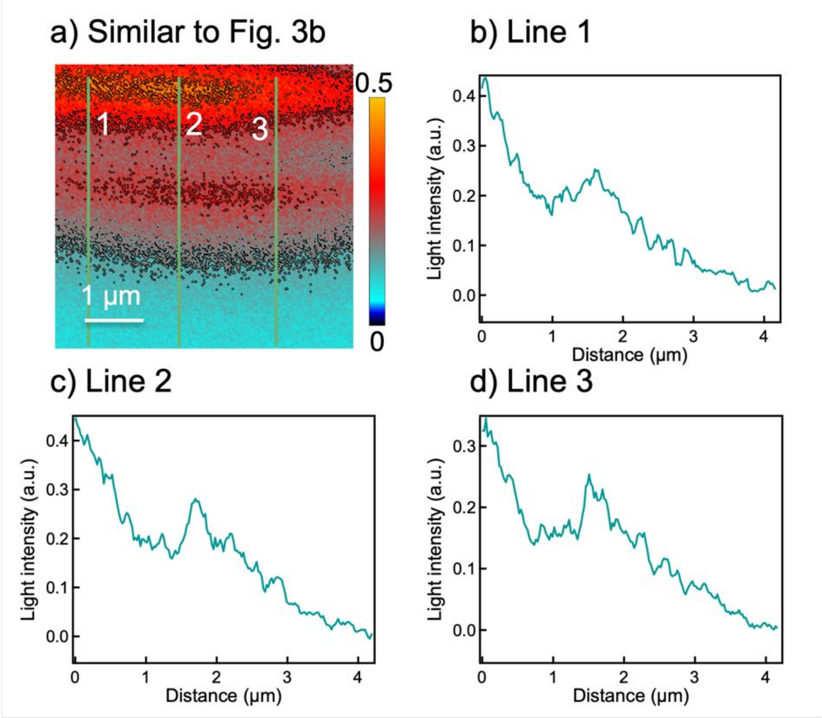

Figure S4. Transport Imaging results on another unstressed device, experimental set up similar to Figure 3b. a) TI mapping; b-d) light intensity profiles of TI marked in a), showing the carrier transport decay profiles are not a single decay.

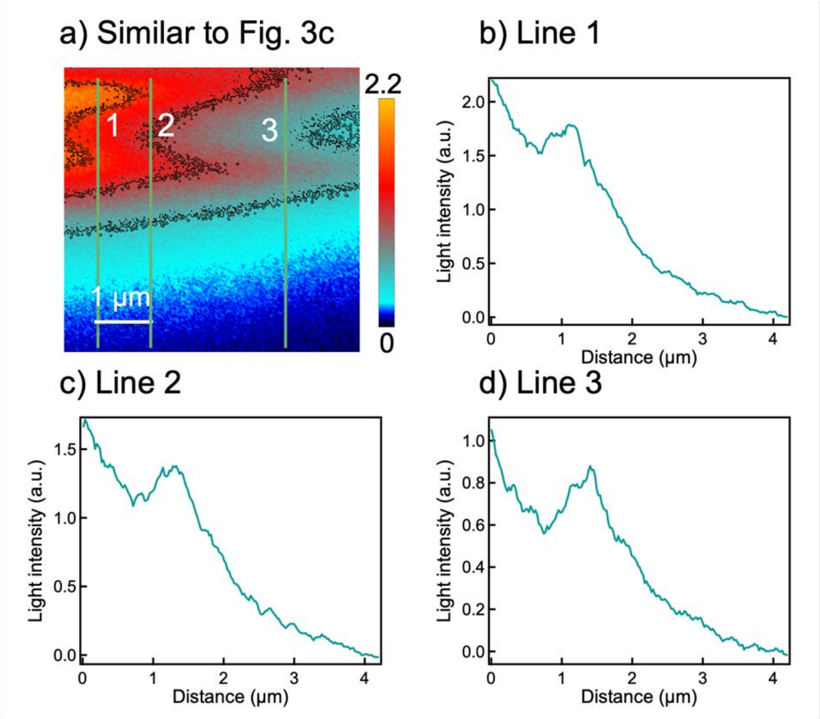

Figure S5. Transport Imaging results on another unstressed device, experimental set up similar to Figure 3c. a) TI mapping; b-d) light intensity profiles of TI marked in a), showing the carrier transport decay profiles are not a single decay.

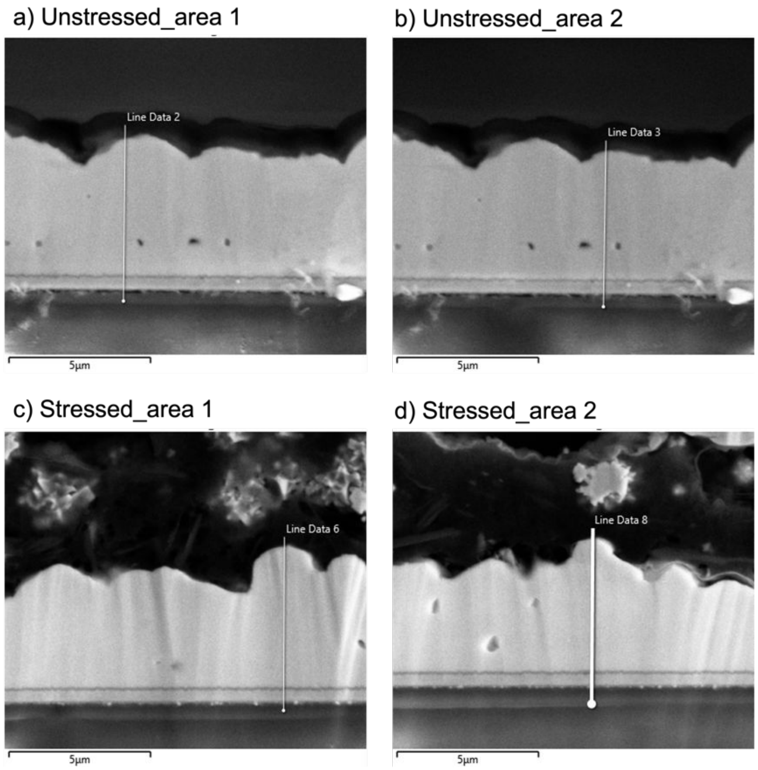

Figure S6. SEM EDS on multiple locations of unstressed and stressed cells, line profiles shown in Figure 5. Note that we chose areas with similar thickness for better comparison. In Figure 5, the shoulder at 3 µm may be due to rough topography, because EDS has a large signal generation volume, the signal at ~3 µm may involve signal from different amounts of underneath materials.

Figure S7 (a) shows the band edges for the two smoothing parameters $s = 0.8$ and $s = 2.4$. The e-beam is located at $d = 1.4$ μm with a generation rate that is sufficient to screen the built-in field near the junction and cause slight fluctuations in the band edges. A zoomed in region of the band edges near the observed TI peaks is shown to illustrate the subtle changes in the energetic differences between the quasi-Fermi levels and their respective bands for the two smoothing parameters. The fluctuations are less pronounced for the $s = 2.4$ case. Electron density profiles corresponding to the band edges are shown in Figure S7(b), exhibiting the two peaks for the $s = 0.8$ case that correspond to the peaks in radiative recombination and the observed TI profiles. There is only a single electron density peak for the $s = 2.4$ case. The hole density profiles follow the same pattern in this region (not shown).

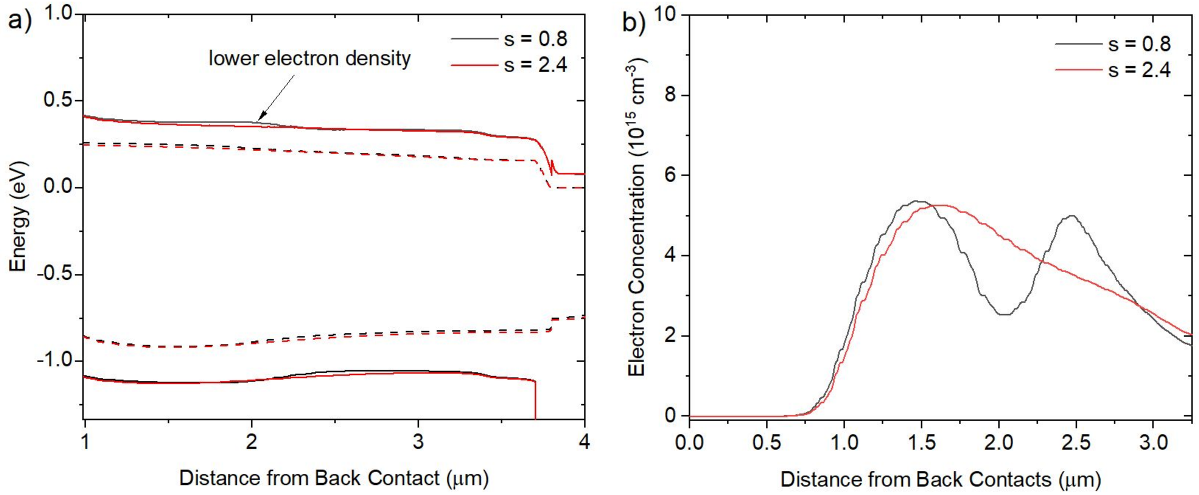

Figure S7. (a) band edges and (b) electron density profiles for e-beam excitation at $d = 1.4$ μm with band gap smoothing parameters of $s = 0.8$ and $s = 2.4$. A close-up of the excitation region shows that at $s = 0.8$, slight fluctuations in the band edges (a) can cause double or single peaks in the electron density (b). A similar result holds for hole density, resulting in the observed peaks in radiative recombination and, therefore, TI measurements.

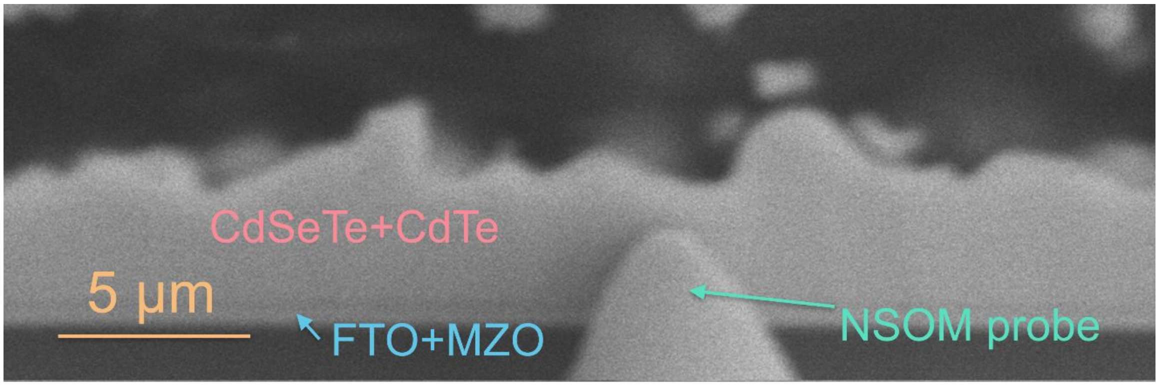

Figure S8. NSOM probe landing on the cross-sectional surface of a CdSeTe/CdTe cell.

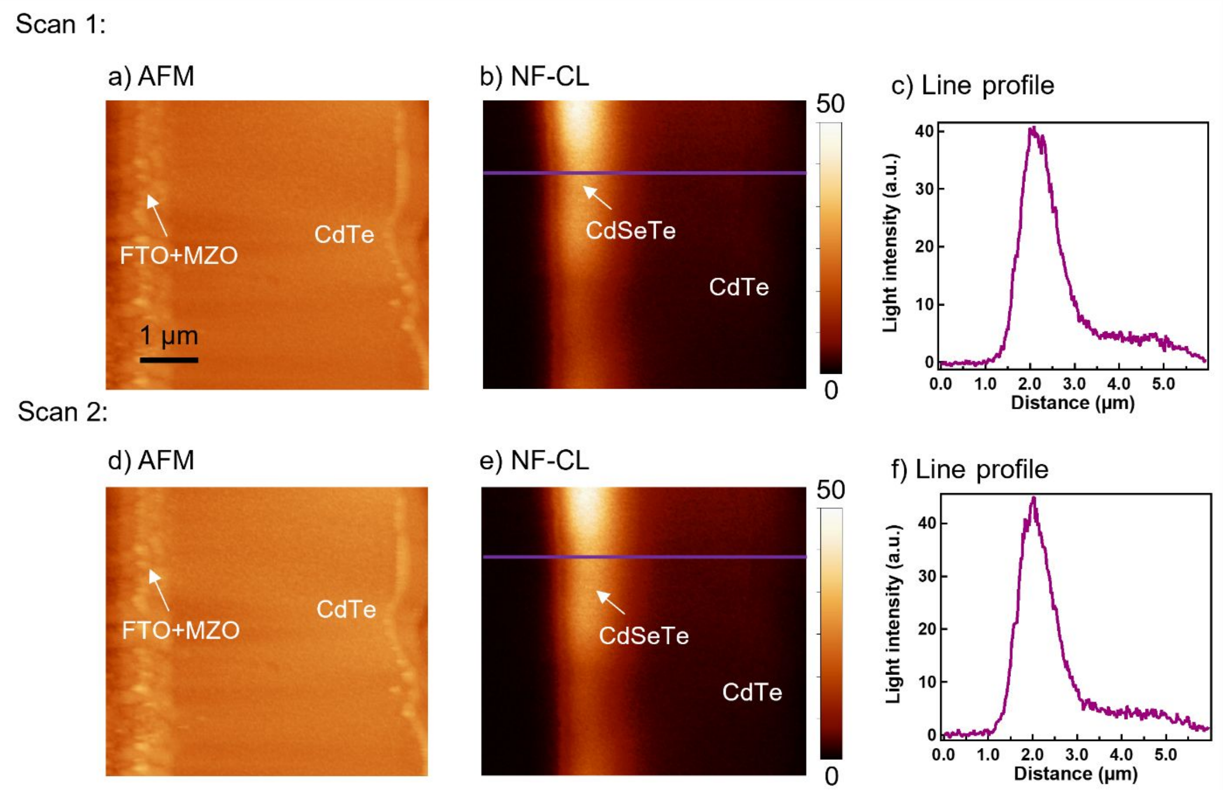

Figure S9. Two consecutive scans of NF-CL on the same location of unstressed CdSeTe/CdTe devices. The electron beam condition is 20 kV, 0.38 nA, which led to a different light intensity than Figure 2. The results demonstrate reliable measurements with no carbon deposition contamination affects the NF-CL and further TI analysis. The AFM data is shown to help visualize where the NF-CL data is coming from.

Table S3. Baseline device model parameters [1–4].

**Parameter**	**Symbol**	**Unit**	**SnO_2:F**	**Mg(Zn,O)**	**p-Cd(Se,Te)**	**n-Cd(Se,Te)**
Thickness	h	nm	400	100	3,400	300
Band Gap	E_g	eV	3.6	3.75	1.5–1.39[a]	1.39
Rel. Permittivity	ε		9.0	9.0	9.4	9.4
Electron Affinity	χ	eV	4.4	4.49[b]	graded[a]	4.49
Mobility	μ_n, μ_p	cm^2/Vs	100, 25	1, 25	320, 40	320, 40
Lifetime	τ_n, τ_p	ns	0.1, 0.1	0.1, 0.1	2, 2	2, 2
Doping	N_A, N_D	cm^{-3}	n: 10^{17}	n: 10^{14}	p: $4x10^{14}$	n: $4x10^{15}$

Notes:

(a) Cd(Se,Te) bandgap was graded as described in main text. The affinity was graded such that the band gap changes were completely accommodated by changes in the conduction band.

(b) MZO electron affinity was reduced to 4.22 eV in the stressed device to account in part for the observed FF loss.

- For the Schottky contacts, front and back metal work functions were set to 4.5 and 5.6 eV (pre-stress)/5.5 eV (post-stress), respectively.
- Surface recombination velocities at the front and back contacts were set to 10^7 cm/s.
- Cd(Se,Te)/MZO interface defect density was 10^9 cm^{-2} acceptor type with capture cross-sections of 10^{-14} cm^2 located at mid-gap.
- Fitting the JV data required adding a series resistance of $R_s = 2\ \Omega\ cm^2$.

References

(1) Munshi, A. H.; Kephart, J.; Abbas, A.; Raguse, J.; Beaudry, J.-N.; Barth, K.; Sites, J.; Walls, J.; Sampath, W. Polycrystalline CdSeTe/CdTe Absorber Cells With 28 mA/cm² Short-Circuit Current. *IEEE Journal of Photovoltaics* **2018**, *8* (1), 310–314. https://doi.org/10.1109/JPHOTOV.2017.2775139.

(2) Kephart, J. M.; McCamy, J. W.; Ma, Z.; Ganjoo, A.; Alamgir, F. M.; Sampath, W. S. Band Alignment of Front Contact Layers for High-Efficiency CdTe Solar Cells. *Solar Energy Materials and Solar Cells* **2016**, *157*, 266–275. https://doi.org/10.1016/j.solmat.2016.05.050.

(3) Gloeckler, M.; Fahrenbruch, A. L.; Sites, J. R. Numerical Modeling of CIGS and CdTe Solar Cells: Setting the Baseline. In *Proceedings of 3rd World Conference on Photovoltaic Energy Conversion, 2003*; 2003; Vol. 1, pp 491-494 Vol.1.

(4) Klein, A. Energy Band Alignment in Chalcogenide Thin Film Solar Cells from Photoelectron Spectroscopy. *J. Phys.: Condens. Matter* **2015**, *27* (13), 134201. https://doi.org/10.1088/0953-8984/27/13/134201.
